# Association of Huntington's disease and schizophrenia-like psychosis in a Huntington's disease pedigree

**DOI:** 10.1186/1745-0179-2-1

**Published:** 2006-02-15

**Authors:** Bernardo Barahona Corrêa, Miguel Xavier, João Guimarães

**Affiliations:** 1 Depart. Psychiatry and Mental Health, Faculty of Medical Sciences – UNL – Calçada da Tapada, 155, 1300-Lisbon, Portugal; 2 Depart. Neurology, Faculty Medical Sciences – UNL, Hospital Egas Moniz, 1400-Lisbon, Portugal

## Abstract

**Background:**

Huntington's disease (HD) is a dominantly inherited, neurodegenerative disorder due to expansion of a polymorphic trinucleotide repeat in the short arm of chromosome 4. Clinical manifestations consist of a triad of choreic movements, cognitive decline and psychiatric syndromes starting in the fourth to fifth decade. Psychiatric manifestations vary and may precede motor and cognitive changes. Personality changes and depression occur most commonly. Paranoid schizophrenia-like symptoms occur in 6% to 25% of cases.

**Case report:**

We describe a 55 year-old woman with an 8 yearlong history of behavioural changes, multi-thematic delusions and auditory hallucinations. History and mental state examination were suggestive of paranoid schizophrenia. Neurological examination revealed discrete, involuntary movements affecting her arms and trunk. Genotyping detected an expanded allele (43 trinucleotide repeats). A three-generation-long family history of chorea and schizophrenia-like psychosis was found.

**Conclusion:**

HD-families have been reported in which schizophrenia-like syndromes emerged in all or most HD-affected members long before they developed extra-pyramidal or cognitive changes. This has been attributed to more than mere coincidence. We hypothesise that in these families the HD gene is transmitted along with a low load of small-effect "psychosis genes" which, in the presence of the severe cognitive changes of HD, manifest as a schizophrenia-like phenotype. Further research is needed in order to clarify the links between genetic loading and the emergence of psychotic symptoms in Huntington's disease.

## Background

Huntington's disease is a dominantly inherited neurodegenerative, neuropsychiatric disorder first described in 1872 by George Huntington. Its prevalence in Caucasian populations is about 1 per 10 000 individuals [1,2]. The genetic mutation underlying this disorder has been localized to the short arm of chromosome 4 and was found in 1993 to consist of an expansion and instability of a polymorphic trinucleotide repeat (CAG repeat) in gene IT15 [3]. Healthy individuals have around 11–35 CAG repeats on this gene, whereas HD patients have 36 or more [4-6]. HD is characterised by selective atrophy of medium spiny neurons in the caudate and putamen and loss of large neurons in the deep layers of the frontal and parietal cortex [1,7]. How the HD mutation leads to these changes is not entirely known. The protein product of the gene, *huntingtin*, contains abnormally long polyglutamine sequences in its mutated form and this is thought to result in the formation of toxic intracellular aggregates. Moreover, wild-type *huntingtin *seems to up-regulate the transcription of neurotrophic factors crucial for neuronal survival and loss of this function is probably central to the neurodegenerative process of HD [8,9]. GABA and acetylcholine are the main neurotransmitters affected in HD. The decrease in cholinergic activity probably explains the deficits in memory retrieval observed in HD (7). Loss of inhibitory GABAergic function and an increased dopamine turnover due to selective survival of type II spiny interneurons has been proposed as an explanation for the emergence of psychotic symptoms in HD (7). HD manifests clinically as a triad of choreic movements, cognitive decline and psychiatric syndromes. Onset usually occurs in the fourth to fifth decade, progressing inexorably to death after 15–20 years [1,10]. An inverse relationship has been consistently demonstrated between the number of CAG repeats and age at disease onset [5,6,10,11]. Affected offspring of HD patients often develop the disease earlier than their parents due to expansion of the inherited abnormal CAG repeat (anticipation). Anticipation seems to occur specially when the CAG repeats are inherited through the male germ line [5,7,11].

Psychiatric and behavioural manifestations of HD are varied both in nature and time of occurrence in the course of the disease, often evolving in the same individual [7,11-13]. Personality changes are the most common behavioural manifestation and probably will occur, eventually, in every patient [1,2,12-15]. Apathy, irritability, aggressive and violent behaviour are the most reported conduct problems. Depression is the second most common psychiatric disorder among HD patients, with a lifetime prevalence of 39%, and most studies report a four- to six fold increase in suicide [1,12-16]. Schizophrenia-like psychosis occurs in HD disease with an estimated frequency of 6% to 25%, the paranoid form being apparently the most common type [1,2,12-14,16]. Patients with an early age at onset of HD seem to have a greater risk of developing psychosis [14,16]. In this report we are especially interested in the association between HD and schizophrenia-like psychotic symptoms and how this may prove relevant to our understanding of the complex genetics of schizophrenia. We describe a family where all known cases of HD developed schizophrenia-like psychosis several years before other manifestations became evident.

## Case report

**Figure 1:**
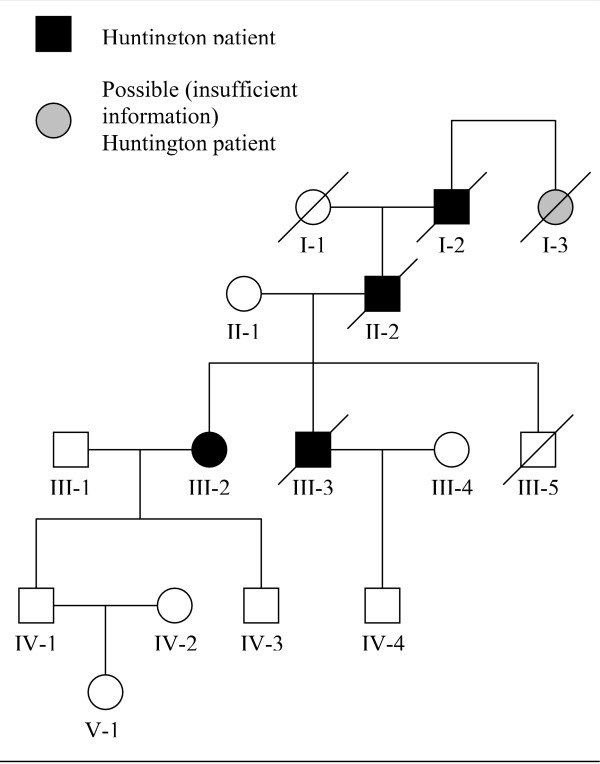
**Family tree**. / – Huntington patient.  – Possible (insufficient information) Huntington patient.

(referred patient is presented firstly, followed by family history; numbers in the headings refer to family tree in fig 1)

### III-2 (referred patient)

This 55-year old retired civil servant was brought by her family to our emergency department. She was acutely agitated and had refused to leave her house or to take any food for the previous two weeks. On mental status examination she was querulous and argumentative, with a slightly irritable mood. She claimed that her youngest son, a police-inspector, had ordered two disguised agents to follow her and insisted that she had recognised them several times in different places. She believed him to be involved in a serious corruption affair as a consequence of which both she and him were soon to be arrested unless they fled the country. She also claimed that a terrible smell came from her mouth and intestines. Although she acknowledged not feeling the smell herself, she was absolutely sure of it, as people would go away from her or shut their windows as she walked through the streets. She seemed to be suffering auditory hallucinations and complained that strangers and neighbours would shout insults at her, even when she was home alone.

According to her family, she had been expressing an increasing concern over the previous 8 years that microphones had been installed in her car by the secret police and that her telephone was under surveillance. These concerns usually were present over periods of several weeks alternating with relatively calmer periods. She had never received psychiatric treatment before.

Her neurological examination revealed a mild motor slowness and very discrete, almost imperceptible involuntary movements affecting her arms and trunk. Brain-CT showed no changes. Mini Mental Status score was 23 in 30. Neuropsychological testing showed difficulty following multi-step instructions, marked compromise of verbal and visual memory, constructive apraxia, and slight difficulties in sustained attention and executive planning. HD genotyping revealed an expanded allele with 43 CAG repeats and an unaffected allele with 19 repeats. Questioning of the available relatives allowed us to put together the family tree shown below. The patient was started on amisulpride up to 600 mg a day, with gradual improvement of her psychotic symptoms and involuntary movements. Cognitive difficulties remained unchanged.

### III-3

This subject became mentally diseased at the age of 40, when he developed persecutory delusions and hallucinations. He went through several admissions to psychiatric hospitals and eventually a diagnosis of schizophrenia was reached. About five years later he progressively developed involuntary limb movements and an unsteady gait. At 49 he was fatally overrun after falling from a sidewalk. No HD genotyping was obtained from him.

### III-5

This subject became heroin-addicted at about 17. He suffered frequent depressive episodes and finally committed suicide at the age of 28. At the time of his death he had never suffered any psychotic episodes or neurological symptoms.

### II-2

This subject was unanimously described as a caring husband and father up to the age of 36, when he unexpectedly divorced his wife under the false belief that she had an extramarital affair going on. Over the years he progressively isolated from his family and friends and lost his job as an agrarian engineer. At 48 schizophrenia was diagnosed and he was admitted to a psychiatric hospital where he remained for several weeks. Over the following years he gradually developed intense involuntary limb movements and cognitive decline, becoming bedridden and dependent on others. He died at 56 from a myocardial infarction.

### I-2

This subject, an army officer, suddenly attacked a general during a field-parade, tearing the latter's insignias away, shouting that he was deliberately sabotaging his career and challenging him to a duel, which in the 1930ies was considered eccentric enough for him to be admitted into a psychiatric hospital. He was then in his early 30ies. Schizophrenia was diagnosed and he eventually was submitted to pre-frontal leucotomy. After this he appears to have become chronically depressed and ended by committing suicide about eight years after the operation. Information is lacking regarding his precise age at the time of operation and his neurological status at any time.

### I-3

Little is known about this subject, except that she became mentally diseased as a young woman. She died in her 60es and was deeply demented by then. It is unknown whether she had other neurological symptoms besides dementia at the time of her death.

## Discussion

In this family Huntington's disease was diagnosed by molecular genetic techniques in only one patient (III-2). In spite of the obvious limitations of collecting clinical data retrospectively, there is enough information to allow a sure diagnosis of HD in subjects III-3 and II-2. Although it is unknown whether subject I-2 was suffering from any signs of chorea at the time he committed suicide, he almost certainly passed the HD mutation on to the next generations, as subject I-1 is known to have died in her mid-80es with no history of psychiatric or neurological disease. Neither IV-1, IV-3, IV-4, nor V-1 had decided to go through HD genotyping at the time of this publication. It is impossible to draw any conclusions regarding subject I-3, due to insufficient information. We thus have a HD-family where all subjects known to have carried the disease also developed schizophrenia-like psychotic symptoms at least five years before neurological or cognitive manifestations became apparent. Single HD-families have been reported where schizophrenia-like psychiatric manifestations emerged in all or most HD-affected members a considerable time before they developed extra-pyramidal or cognitive changes [17-19]. This is in contrast with the usual psychiatric manifestations of HD, which tend to be highly variable within the same individual, emerge shortly before or coincidentally with the onset of chorea and consist predominantly of non-psychotic syndromes. Psychotic manifestations are usually rare and non-systematized, and occur mostly in patients who are already demented [12,13,20]. Such observations have raised the question of whether certain families might develop schizophreniform psychosis as a specific manifestation of a subtype of HD.

Several hypotheses have been proposed. One possibility would be that the children of HD patients are prone to higher rates of psychiatric disorders in general as a consequence of familiar disruption and the impending risk of developing the disease themselves [1,15,21-24]. Some studies did demonstrate a higher prevalence of adjustment reactions, antisocial conduct or personality disorder, and neurotic disorders among the offspring of HD patients, irrespective of gene-status. Prevalence rates of schizophrenia, however, were not significantly increased [1,21,23-25].

Another possibility is that in these families the HD gene might causes both the extra-pyramidal/cognitive decline syndrome and the schizophreniform disorder [17-19]. This obviously means that psychotic and non-psychotic HD families would have to carry different HD genes, which until now remains to be demonstrated [17-19]. CAG repeat length does not differ consistently between psychotic and non-psychotic HD patients [6,17,18,20].

Still another possibility is that psychosis in these families results from the co-occurrence of the HD gene and a pro-schizophrenia gene or group of genes. The hypothesis of a single gene in linkage disequilibrium with the HD gene has not been demonstrated by research [17,19,26]. An explanation more in hand with the known oligogenic nature of "primary" schizophrenia is that the HD gene could lower the threshold for the emergence of a schizophrenic phenotype [18,19]. Put otherwise, in the presence of a low load of small effect schizophrenia genes the HD gene may behave as a large effect schizophrenia gene. Although large effect schizophrenia genes or candidate genes have not been identified, they have been postulated to occur in high density, multiply affected schizophrenia families [26]. A similar explanation has been proposed by Holmes et al for their finding that variation in dopamine receptor DRD3 (a candidate small effect gene of schizophrenia) is associated with development of delusions in Alzheimer Disease patients [27]. Assuming that schizophrenia oligogenes are by definition more frequent in the general population than schizophrenia itself, this would explain why schizophrenia is more frequent among HD carriers than in the general population [18]. In fact, if the co-occurrence of schizophrenia and HD was merely due to a coincidental accumulation of phenotypes, then the exact opposite should be expected. For some time, family and linkage studies on Schizophrenia and Bipolar Disorder suggested that these disorders might constitute two possible phenotypes of the same polygenic disorder. After this apparent rebirth of unitary psychosis, however, it is now becoming increasingly clear that the two disorders share some, albeit not all of their genetic determinants [28-31]. Some authors suggest that the two diseases might share one or more "psychosis genes". Interaction of this common "psychotic background" with specific pro-schizophrenia or pro-bipolar genes would then produce either Schizophrenia or Bipolar Disorder with psychotic symptoms, respectively [28,29,32,33]. The possible aggregation of psychotic symptoms in certain HD families may shed some light on how these hypothetical pro-schizophrenia genes may act. If the "psychosis genes" correspond phenotypically to the clinical dimension hallucinations/delusions, then it is possible that in these families the HD gene may act as a pro-schizophrenia gene. This raises the interesting hypothesis that pro-schizophrenia genes might determine some form of subcortical dysfunction responsible for the dimensions thought disorganization/negative symptoms. Association with the hallucinations/delusions phenotype of the unspecific pro-psychosis genes would then give rise to the full Schizophrenia syndrome. It is highly relevant, in this regard, the demonstration of significantly higher thought disorder scores among biological siblings and half-siblings of adopted schizophrenic probands when compared to the siblings of adopted healthy probands, with similarly low rates observed among the adoptive relatives of both groups [34].

## Conclusion

Only very few cases of single HD-families have been reported where schizophrenia-like psychiatric manifestations emerged in all or most HD-affected members long before they developed motor or cognitive changes [17-19].

Such families may prove a valuable models in our understanding of the genetics of non-organic psychosis, namely of schizophrenia.

It must be stressed that these considerations are based on the empirical impression that psychotic symptoms tend to aggregate in certain HD families. This assumption is based merely on isolated reports of single families and has not been demonstrated objectively. Further research is needed in order to clarify the links between genetic loading and the emergence of psychotic symptoms in Huntington's disease.

## Competing interests

The author(s) declare that they have no competing interests.

## Authors' contributions

BC wrote and submitted the manuscript

MX helped to draft the manuscript and reviewed published references

JG revised the manuscript critically from the neurological point of view

The authors read and approved the final manuscript. The authors were involved in the care of the patient described in this case report
